# [Retracted] Classical dendritic cells regulate acute lung inflammation and injury in mice with lipopolysaccharide-induced acute respiratory distress syndrome

**DOI:** 10.3892/ijmm.2026.5884

**Published:** 2026-06-08

**Authors:** Lang Li, Liang Dong, Dan Zhao, Fei Gao, Jie Yan

Int J Mol Med 44: 617-629, 2019; DOI: 10.3892/ijmm.2019.4208

Following the publication of this paper, it was drawn to the Editor's attention by a concerned reader that, regarding the lung tissue images shown in Fig. 5B on p. 624, the 'FLT3L+AARDS' and 'DMSO+AARDS' panels contained an overlapping section of data, such that data which were intended to show the results of differently performed experiments had apparently been derived from the same original source. Although the possibility of publishing a corrigendum was considered, upon performing an independent analysis of the data in this paper in the Editorial Office, it came to light that western blot data in Fig. 1A and D had previously been published in a pair of papers in the journal *PloS One* which were written by different authors at different research institutes.

Owing to the fact that the contentious western blot data in the above article were found to be strikingly similar to data that had already been published elsewhere, the Editor of *International Journal of Molecular Medicine* has decided that this paper should instead be retracted from the Journal. The authors were asked for an explanation to account for these concerns, but the Editorial Office did not receive a reply. The Editor apologizes to the readership for any inconvenience caused.

